# Quasi-In Situ Observation of the Microstructural Response during Fatigue Crack Growth of Friction Stir Welded AA2024-T4 Joint

**DOI:** 10.3390/ma17092106

**Published:** 2024-04-29

**Authors:** Jun Yang, Xianmin Chen, Huaxia Zhao, Jihong Dong, Feng Jin

**Affiliations:** 1 National Key Laboratory of Strength and Structural Integrity, AVIC Aircraft Strength Research Institute, 86 2nd Dianzi Road, Xi’an 710065, China; vitochan@163.com; 2 The 3rd Department, AVIC Aircraft Strength Research Institute, 86 2nd Dianzi Road, Xi’an 710065, China; 3 AVIC Manufacturing Technology Institute, Beijing 100024, China; zhaohuaxia@cfswt.com (H.Z.); zhhq@sina.com (J.D.); 4 School of Materials Science and Engineering, Jiangsu University, Zhenjiang 212013, China

**Keywords:** friction stir welding, microstructural response, fatigue crack growth, AA2024-T4, EBSD

## Abstract

The reliability of friction stir welded joints is a critical concern, particularly given their potential applications in the aerospace manufacturing industry. This study offers a quasi-in situ observation of the microstructural response during fatigue crack growth (FCG) of a friction stir welded AA2024-T4 joint, aiming to correlate fatigue crack growth behavior with mechanical properties investigated using electron backscatter diffraction (EBSD). Notched compact tension (CT) specimens corresponding to the morphology of the stir zone (SZ), advancing side (AS), and retreating side (RS) were meticulously designed. The findings indicate that the welding process enhances the joint’s resistance to fatigue crack growth, with the base metal exhibiting a shorter fatigue life (i.e., ~10^5^ cycles) compared to the welding zones (SZ ~ 3.5 × 10^5^ cycles, AS ~ 2.5 × 10^5^ cycles, and RS ~ 3.0 × 10^5^ cycles). Crack propagation occurs within the stir zone, traversing refined grains, which primarily contribute to the highest fatigue life and lowest FCG rate. Additionally, cracks initiate in AS and RS, subsequently expanding into the base metal. Moreover, the study reveals a significant release of residual strain at the joint, particularly notable in the Structural-CT-RS (Str-CT-RS) sample compared to the Str-CT-AS sample during the FCG process. Consequently, the FCG rate of Str-CT-AS is higher than that of Str-CT-RS. These findings have significant implications for improving the reliability and performance of aerospace components.

## 1. Introduction

Friction stir welding (FSW) has emerged as the preferred method for assembling aluminum alloys, owing to its manifold benefits, such as defect reduction and property enhancement [1,2,3]. FSW achieves joint formation through a combination of frictional heat and plastic deformation, resulting in distinct regions within the joint: the stir zone (SZ), advancing side (AS), retreating side (RS), and base metal (BM) [4,5,6]. The SZ represents the central region where the FSW tool interacts with the workpieces, undergoing significant plastic flow and grain refinement to form a metallurgical bond [7]. The AS and RS refer to the sides of the joint where the FSW tool moves forward and backward, respectively, during welding, with the AS experiencing greater plastic deformation and thermal effects [8,9]. These zones are crucial focal points in the study of FSW due to their varying thermal–mechanical effects.

The reliability of FSW joints is particularly crucial for aerospace applications [10], with research traditionally focused on microstructural and tensile properties [11,12,13]. For instance, Malopheyev et al. [11] noted that higher welding speeds improved the uniformity of precipitation distribution in the SZ during subsequent aging, enhancing joint performance. Huang et al. [12] investigated the microstructure, hardness, and tensile characteristics of AA5083 friction stir welded joints, observing diminished mechanical qualities post-welding, particularly in the heat-affected zone (HAZ), which exhibited reduced tensile strength and elongation compared to the BM.

Subsequent studies delved into fatigue performance [14,15,16], with Takao et al. [14] analyzing the fatigue life of non-through cracks in friction stir welded AA2024-T3 joints, noting the influence of welding conditions on fracture locations. As research progressed, fatigue crack growth (FCG) behavior became a prominent topic [17,18,19,20]. Notched compact tension (CT) specimens were employed to simulate FCG behavior at different joint regions. For instance, Vuherer et al. [17] investigated FCG behavior in FSW AA2024-T351 joints, revealing no significant differences between welding speeds. Zhang et al. [18] examined the microstructural and mechanical evolution of FSW AA6061-T6 joints, evaluating the impact of notch locations on FCG behavior and highlighting the influence of longitudinal residual stresses and microstructural inhomogeneity. However, their study oversimplified the distinctions in microstructure between the AS and RS by considering them solely as the heat-affected zone (HAZ). Additionally, the conventional compact tension (CT) samples, with notches perpendicular to the direction of the plate, typically used to investigate FCG behavior, fail to adequately capture the influences of microstructures at the AS and RS on crack growth behavior. This limitation arises due to the angled orientation of the AS and RS relative to the plate direction. Simultaneously, there has been insufficient attention devoted to the observation and correlation of fatigue crack growth behavior with mechanical properties.

Addressing these research gaps, this study focuses on AA2024-T4 friction stir welded joints, employing notched CT specimens corresponding to SZ, AS, and RS morphologies to analyze microstructural responses during the FCG process. Fatigue crack growth performances at various regions were characterized using the Paris model, with electron backscatter diffraction (EBSD) employed to examine microstructures along the crack propagation path. The study facilitates the quasi-in situ observation of microstructural responses during fatigue crack growth, providing insights into crack growth behavior and contributing to the reliability of FSW joints, particularly in aerospace applications.

## 2. Experimental Procedures

### 2.1. Friction Stir Welding

**Figure 1:**
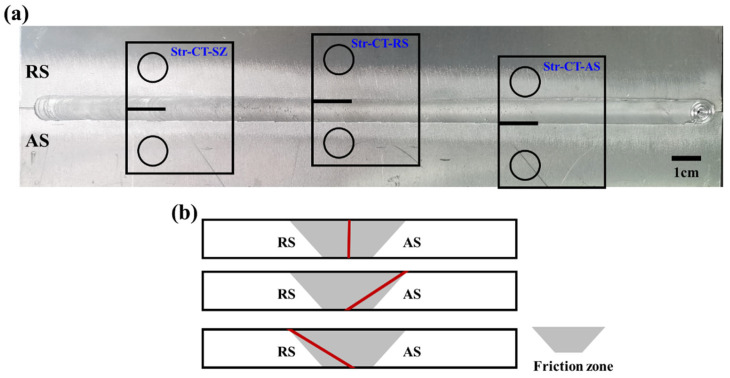
The macro morphology of an FSW AA2024-T4 joint with a schematic showing of CT samples: (**a**) the macro morphology and the notch locations and (**b**) the schematic showing of the microstructural notch according to the joint morphology.

The base material comprised 2024-T4 aluminum alloy plates measuring 300 mm × 100 mm × 2 mm. Prior to welding, thorough cleaning ensured consistent and high-quality welds. The plates were joined using a rotation speed of 1200 rpm and a traverse speed of 300 mm/min [21] by a specialized FSW machine (FSW-RL31-010, Beijing FSW Technology Co., Ltd., Beijing, China). In Figure 1, the macro morphology of the friction stir welded (FSW) joint is illustrated, with a schematic depiction of the CT samples. Notably, Figure 1a highlights that the AS typically exhibits a smoother surface finish compared to the RS. The smoother surface finish observed on the AS compared to the RS in FSW can be attributed to the direction of tool movement [7,8,9]. The tool moves forward on the AS, resulting in more uniform plastic deformation and thermal effects, which can lead to a smoother surface. Conversely, on the RS, where the tool moves backward or retreats, there may be more irregularities in plastic deformation and thermal effects, potentially resulting in a rougher surface finish. Subsequent to the welding experiment, samples for FCG testing were prepared. Figure 1a illustrates the notch locations, while Figure 1b schematically presents the microstructural notches corresponding to the joint morphology.

### 2.2. Fatigue Crack Growth Test

**Figure 2:**
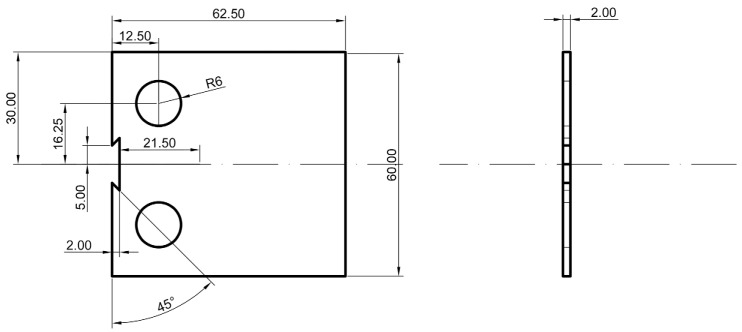
Sample geometry for fatigue crack growth rate tests (in mm).

The FCG tests were conducted in accordance with the ASTM E647 standard [22]. Figure 2 illustrates the detailed dimensions of the CT samples utilized. All FCG tests were performed at room temperature with a stress ratio (R) of 0.1 and a frequency of 10 Hz. The maximum load (Pmax) was set at 1000 N, while the minimum load (Pmin) was maintained at 100 N. The FCG direction aligned parallel to the welding direction, with four groups of CT samples prepared, each featuring a crack tip notched at different regions: BM, SZ, AS, and RS, respectively. To emphasize the impact of various microstructures within the SZ, AS, and RS of the friction stir welded joint on FCG behavior, the samples were designated as follows: CT-BM, Structural-CT-SZ (Str-CT-SZ), Structural-CT-AS (Str-CT-AS), and Structural-CT-RS (Str-CT-RS), as depicted in Figure 1. Crack length was continuously measured using the compliance method with a crack mouth clip gauge. Data on the number of cycles and crack length were collected for further analysis.

The analysis of FCG behavior is based on the classic Paris model, a widely used framework in FCG testing [23], originally introduced by Paris and Erdogan [24]. Thus, fatigue crack growth behavior was examined utilizing power law Equations (1) and (2) [18], while for compact tension (CT) samples, the stress intensity factor range (∆K) was determined using Equation (3) [25].

(1) $$\frac{da}{dN}=C{\left(∆K\right)}^{m}$$

(2) $$\mathrm{lg}\left(\frac{da}{dN}\right)=mlg∆K+lgC$$

(3) $$∆K=\frac{∆P}{B\sqrt{W}}\frac{\left(2+a\right)}{{\left(1-a\right)}^{\frac{3}{2}}}(0.886+4.64\alpha -13.32\alpha^{2}+14.72\alpha^{3}-5.6\alpha^{4})$$

where, α=a/W; ∆P is the variation of the load; *B* is the thickness of the C(T) sample, mm; *W* is the width of the C(T) sample, mm; m, C are the material parameters.

(4) $${\left(da/dN\right)}_{\bar{a}}=(a_{i+1}-a_{i})/(N_{i+1}-N_{i})$$

Thereafter, relationship of FCG rate da/dN and the stress intensity factor range ∆K in stable propagation stage can be obtained.

### 2.3. EBSD Examination

To achieve quasi-in situ observation of the microstructural response during fatigue crack growth of friction stir welded AA2024-T4 joints, EBSD was utilized to examine and characterize the distinctive zones. Initially, the joint underwent scanning, and the microstructures at the SZ, AS, and RS were characterized. Subsequently, along the FCG path, the relevant zones were scanned with EBSD. To distinguish the FCG behavior of AS and RS, the microstructural response of the joint during the crack propagation process at AS and RS was observed using EBSD. All EBSD samples underwent polishing by a mechanical polishing machine followed by electro-polishing in a solution consisting of 30 mL HClO_4_ and 270 mL CH_3_CH_2_OH at 0~5 °C for 15~20 s. EBSD analysis was conducted using the OXFORD NORDLY MAX3 system and HKL-Channel5 software. The step size for the EBSD scan was set to 0.2 µm, operating at 20 kV with an inclination angle of 70 degrees. Inverse pole figure (IPF) maps were utilized to characterize the microstructures, with boundaries between grains having different orientations of 15° or higher defined as high-angle boundaries. Additionally, kernel average misorientation (KAM) maps were employed to analyze the local strain within the joint.

## 3. Results

### 3.1. Microstructure of the Joint

**Figure 3:**
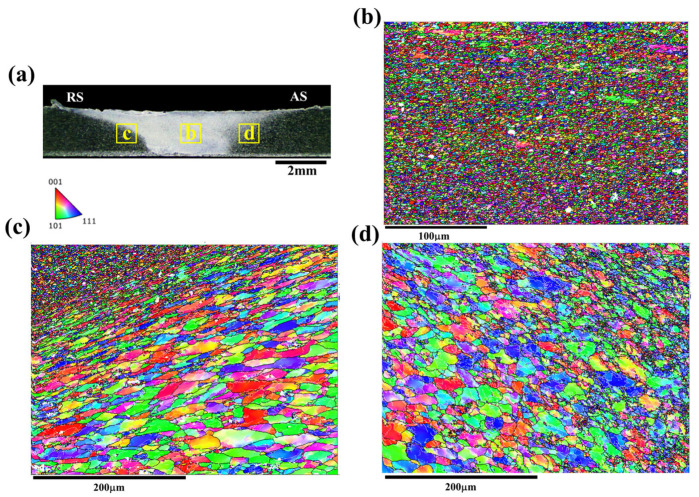
The IPF maps showing the microstructure of FSW AA2024 joint: (**a**) the morphology, (**b**) the microstructure of SZ, (**c**) the microstructure of RS and (**d**) the microstructure of AS.

The microstructure of the FSW AA2024 joint is depicted in Figure 3, where Figure 3a illustrates the overall morphology of the joint, while Figure 3b–d present the inverse pole figure (IPF) maps of the SZ, AS, and RS, respectively. In the SZ, as shown in Figure 3b, a refined microstructure is observed due to dynamic recrystallization. The rotating tool induces severe plastic deformation and frictional heating, leading to the breakdown and reorientation of the grains. Consequently, dynamic recrystallization occurs, resulting in the formation of refined equiaxed grains. In the RS, depicted in Figure 3c, the microstructure displays evidence of deformation, such as elongated grains or flow patterns, caused by the movement of the FSW tool away from the welding direction. This side experiences lower temperatures and less severe plastic deformation compared to the AS [7,8,9]. Conversely, the AS shown in Figure 3d exhibits some degree of deformation and flow patterns, albeit to a lesser extent compared to the RS. The AS experiences higher temperatures and more intense plastic deformation, resulting in grains that are typically equiaxed, closely resembling the grain structure of the base metal. Unlike the RS, the AS zone does not display prominent flow patterns resulting from plastic deformation and mixing.

### 3.2. FCG Behavior at Different Regions

**Figure 4:**
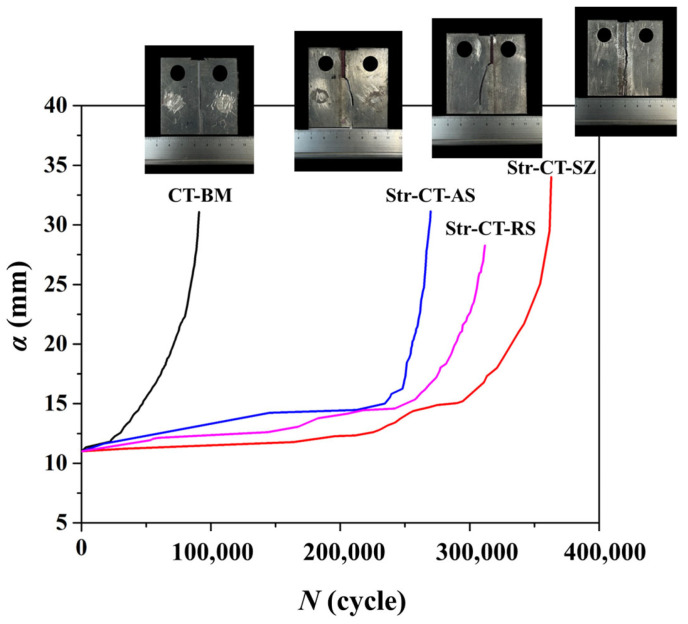
The relationship of fatigue crack length *a* and cycle index *N*.

The FCG behavior at different regions, namely the BM, SZ, AS, and RS, can be influenced by microstructural variations, local stress concentrations, or specific welding conditions characteristic of each zone [26,27]. This study focuses on correlating FCG behavior and mechanical properties with a quasi-in situ observation of microstructural responses. Figure 4 illustrates the measured crack lengths plotted against the counted cycles for various cases. The results indicate that the base metal (CT-BM) exhibits a shorter fatigue life (i.e., lower fatigue cycles, ~10^5^ cycles) compared to the welding zones (SZ ~ 3.5 × 10^5^ cycles, AS ~ 2.5 × 10^5^ cycles, and RS ~ 3.0 × 10^5^ cycles), suggesting greater resistance to crack propagation in the welding zones. Among the SZ, AS, and RS, SZ (i.e., Str-CT-SZ) demonstrates the highest fatigue load cycle. Furthermore, it is observed that the SZ, AS, and RS exhibit similar FCG behavior compared to the BM. The smooth and continuous crack growth curve for CT-BM suggests stable crack propagation behavior. In contrast, the curves for Str-CT-SZ, Str-CT-AS, and Str-CT-RS display noticeable stages, which will be discussed and characterized using the Paris model in the subsequent analysis.

**Figure 5:**
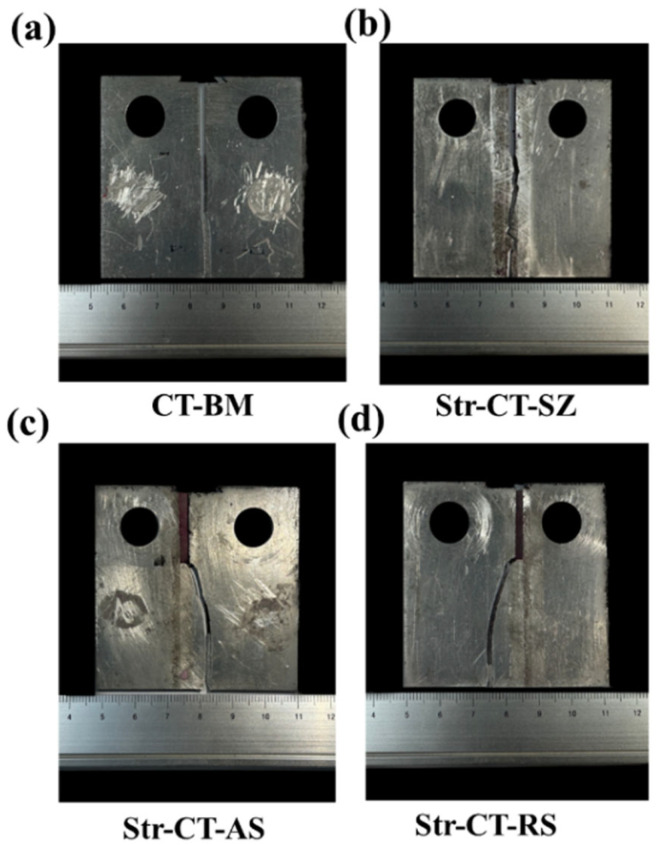
The morphologies of CT samples after FCG test: (**a**) the morphology of CT-BM, (**b**) the morphology of Str-CT-SZ, (**c**) the morphology of Str-CT-AS and (**d**) the morphology of Str-CT-RS.

Figure 5 depicts the morphologies of CT samples after the FCG test. In Figure 5a, the BM displays characteristics of straight crack propagation. Conversely, Figure 5b illustrates the tortuous path of crack propagation in the SZ. Cracks in the AS and RS regions exhibit similar behavior, initiating at the AS or RS and then expanding to the BM, as shown in Figure 5c and Figure 5d, respectively. The microstructure along the fatigue crack propagation path will be characterized in Section 4.1.

**Figure 6:**
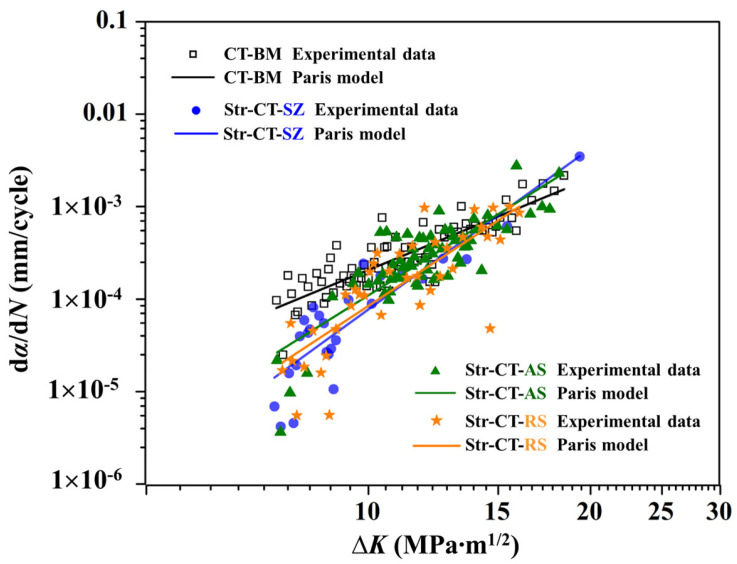
FCG rate curves described by experimental data and Paris model.

The FCG process is typically divided into three stages: low-speed propagation stage, stable propagation stage, and fast propagation stage. Figure 6 presents the relationship between the FCG rate da/dN and the stress intensity factor range ∆K in the stable propagation stage, based on Equations (1)–(4), which are commonly employed in FCG testing, as introduced by Paris and Erdogan. The fitting results exhibit a linear relation in the stable propagation stage. Initially, the FCG rate follows the order CT-BM > Str-CT-AS > Str-CT-RS > Str-CT-SZ, reflecting the influence of microstructure and longitudinal stress parallel to the welding direction [18]. The FSW process enhances material resistance to FCG, as evidenced by the lowest FCG rate in the SZ due to refined grains, while cracks in the AS and RS propagate from prefabricated positions to the BM. Moreover, compressive longitudinal stresses in the SZ retard fatigue crack growth, whereas tensile stresses in the BM, AS, and RS are the primary reason for cracking. Notably, the slightly higher FCG rate of Str-CT-AS compared to Str-CT-RS warrants further discussion regarding the microstructural response during crack propagation in the AS and RS. At high ∆K, the FCG rate in the joint zone (i.e., the SZ, AS, and RS) exceeds that of the BM, potentially attributable to metallurgical defects in the joint [18]. Table 1 summarizes the fitting parameters and corresponding modeling equations for CT samples to distinguish FCG behaviors of the BM, SZ, AS, and RS using the Paris model, presented as Equations (5)–(8).

The Paris models:

(5) $$\frac{da}{dN}=1.05\times 10^{-7}{\left(∆K\right)}^{3.29}\;(\mathrm{BM})$$

(6) $$\frac{da}{dN}=1.20\times 10^{-10}{\left(∆K\right)}^{5.79}\;(\mathrm{SZ})$$

(7) $$\frac{da}{dN}=1.12\times 10^{-9}{\left(∆K\right)}^{4.98}\;(\mathrm{AS})$$

(8) $$\frac{da}{dN}=4.89\times 10^{-10}{\left(∆K\right)}^{5.25}\;(\mathrm{RS})$$

## 4. Discussion

### 4.1. Observation of the Microstructure at FCG Path

**Figure 7:**
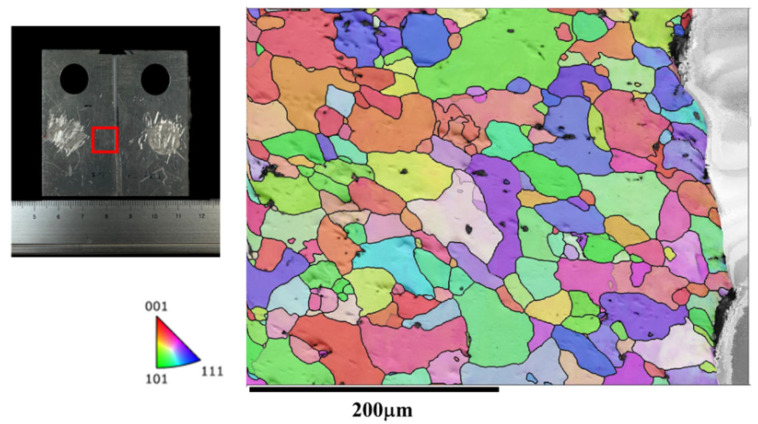
The IPF map of the microstructure at the crack of the BM after the FCG test.

**Figure 8:**
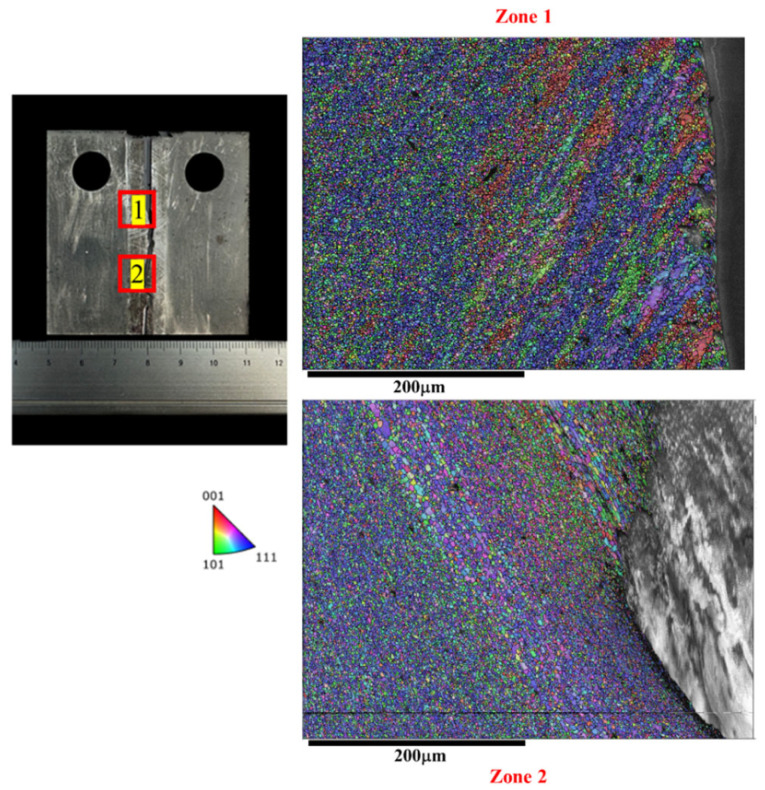
The IPF map of the microstructure at the crack of the SZ after the FCG test.

In Figure 7, the microstructure along the crack path during the crack growth process in the BM is characterized by EBSD (i.e., The red box marks the EBSD zone). The crack expansion in the base material exhibits a straight path, which is typical. The BM displays a uniform microstructure throughout, unaffected by FSW. This consistent microstructure correlates with a lower fatigue life and higher FCG rate compared to the joint microstructure. Figure 8 illustrates the microstructure along the tortuous crack path in the SZ. The results reveal that the fatigue crack passes through the refined grains in the SZ, primarily responsible for the highest fatigue life (as shown in Figure 4) and lowest FCG rate (as shown in Figure 6) of the Str-CT-SZ sample.

**Figure 9:**
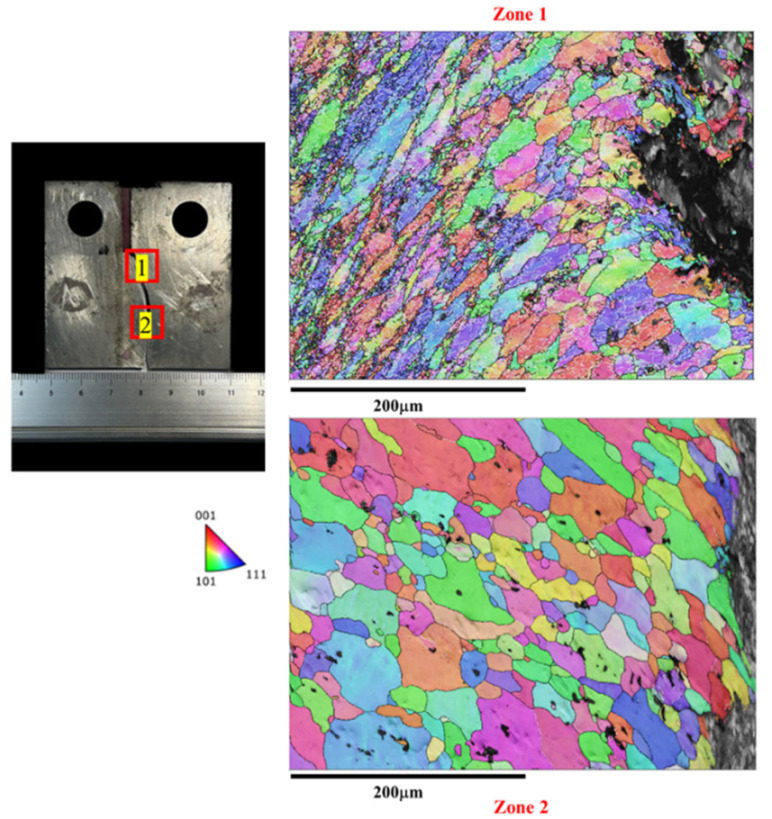
The IPF map of the microstructure at the crack of the AS after the FCG test.

**Figure 10:**
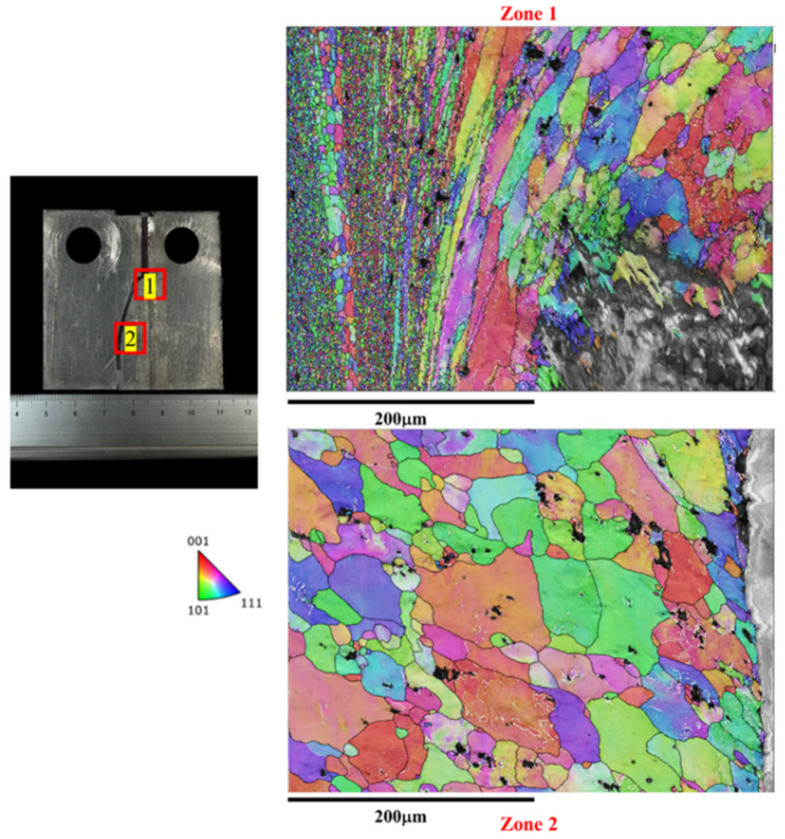
The IPF map of the microstructure at the crack of the RS after the FCG test.

On the other hand, Figure 9 and Figure 10 depict the microstructure along the crack path in the AS and RS, respectively. Both the Str-CT-AS and Str-CT-RS samples show cracks propagating from the prefabricated position to the base material. This suggests that the fatigue crack growth resistance of the welding zone (SZ, AS, RS) is higher than that of the BM. These results offer an insightful view and quasi-in situ observation of the microstructural response along the crack path during the FCG process, providing more detailed microstructural information beyond the macro morphologies shown in Figure 5.

### 4.2. Microstructural Response of the Joint during the FCG Process at the AS and RS

**Figure 11:**
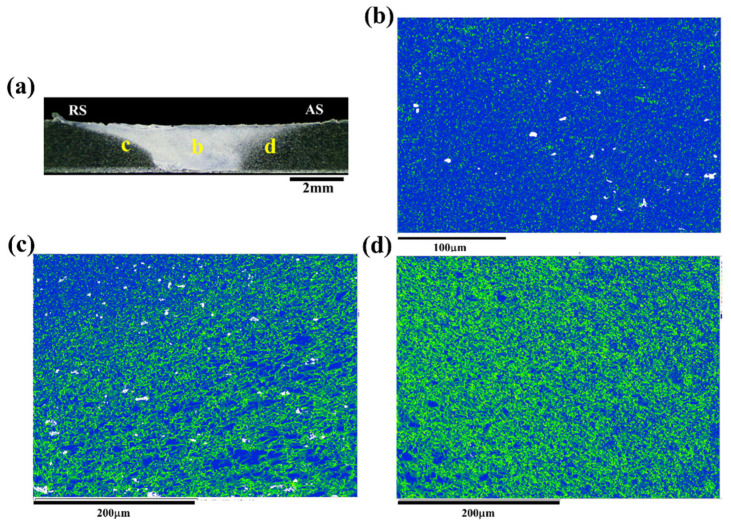
The KAM maps showing the residual strain of the FSW AA2024 joint: (**a**) the morphology, (**b**) the KAM map of the SZ, (**c**) the KAM map of the RS, and (**d**) the KAM map of the AS.

**Figure 12:**
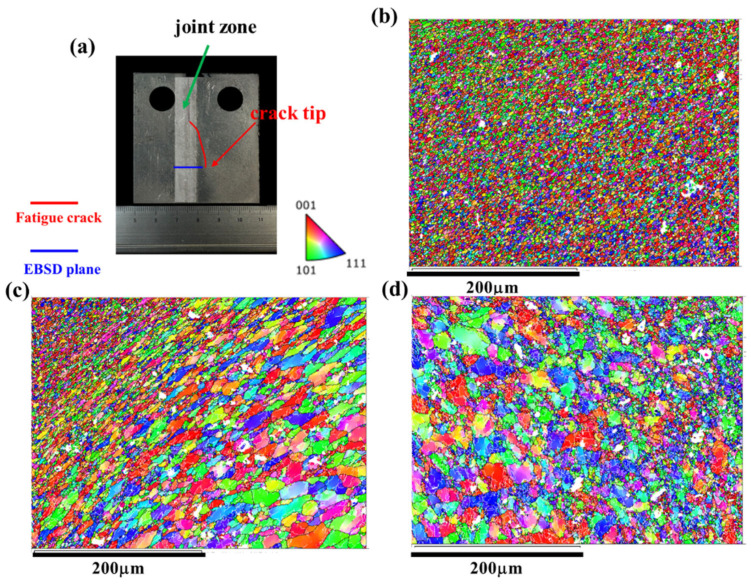
The IPF maps showing the microstructural response of the joint corresponding to the crack tip at the AS: (**a**) schematic showing of the fatigue crack and EBSD plane, (**b**) the microstructure of the SZ, (**c**) the microstructure of the RS, and (**d**) the microstructure of the AS.

**Figure 13:**
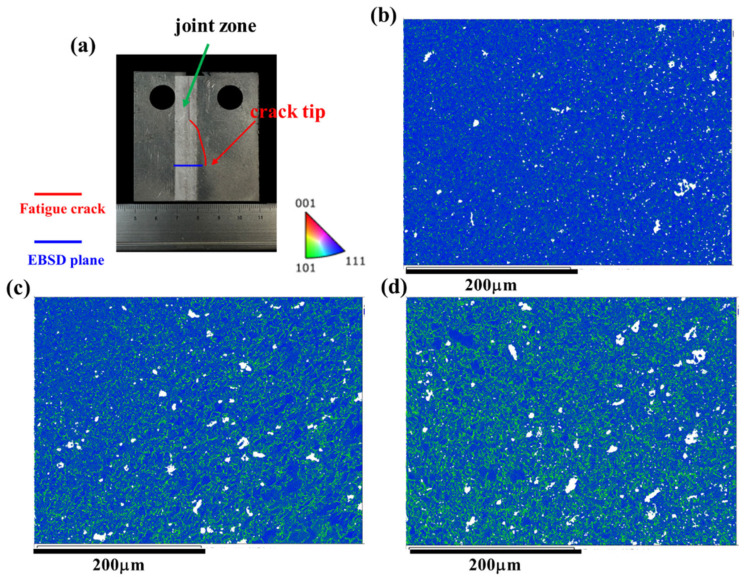
The KAM maps showing the microstructural response of the joint corresponding to the crack tip at the AS: (**a**) schematic showing of the fatigue crack and EBSD plane, (**b**) the microstructure of the SZ, (**c**) the microstructure of the RS, and (**d**) the microstructure of the AS.

**Figure 14:**
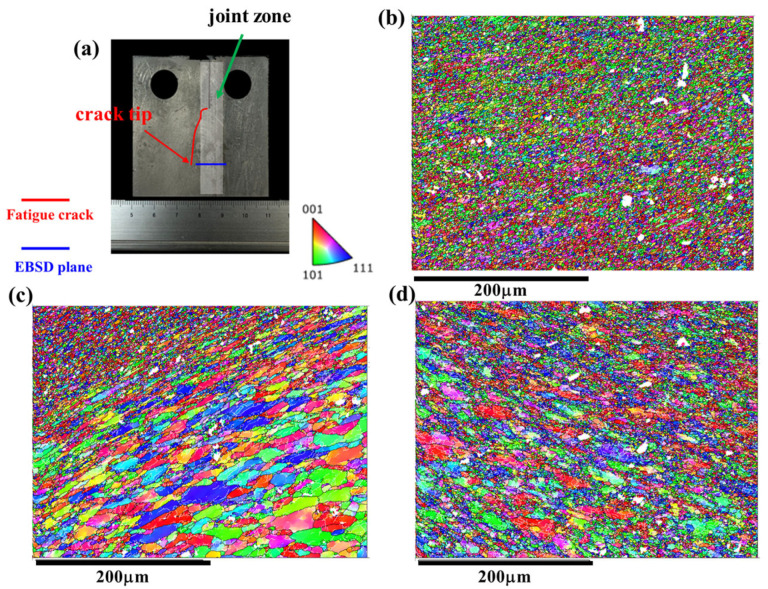
The IPF maps showing the microstructural response of the joint corresponding to the crack tip at the RS: (**a**) schematic showing of the fatigue crack and EBSD plane, (**b**) the microstructure of the SZ, (**c**) the microstructure of the RS, and (**d**) the microstructure of the AS.

**Figure 15:**
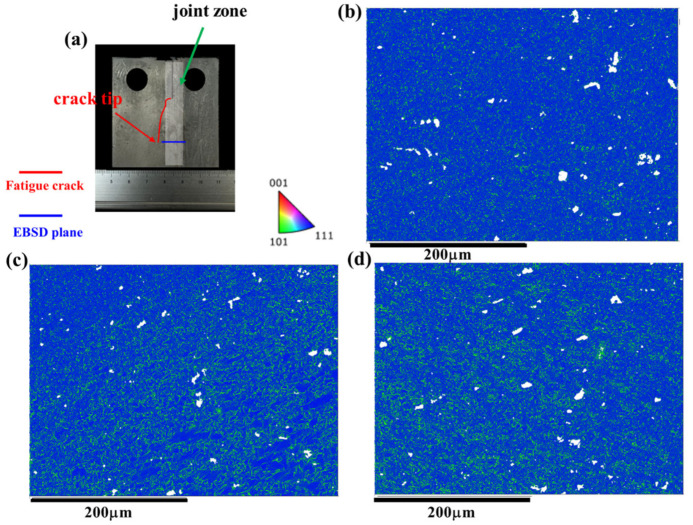
The KAM maps showing the microstructural response of the joint corresponding to the crack tip at the RS: (**a**) schematic showing of the fatigue crack and EBSD plane, (**b**) the microstructure of the SZ, (**c**) the microstructure of the RS, and (**d**) the microstructure of the AS.

As discussed above, the microstructure along the crack path in the AS as depicted in Figure 9 and the RS as shown in Figure 10 exhibit similar features. However, it is noteworthy that the FCG rate of the Str-CT-AS sample is slightly higher than that of Str-CT-RS, as indicated in Figure 6. This suggests that residual strain can also influence the FCG rate, in addition to microstructural factors such as grain size and distribution [18]. Figure 11 presents the kernel average misorientation (KAM) maps illustrating the residual strain of the FSW AA2024 joint. The results reveal that residual strain is predominantly concentrated in the AS (as shown in Figure 11d) and RS (as shown in Figure 11c) compared to the SZ (as shown in Figure 11b). To further distinguish the FCG rate of the AS and RS, stop-action during the FCG test was employed to capture the microstructural response during the FCG process when the crack extended to two-thirds of its final length, as demonstrated in Figure 12, Figure 13, Figure 14 and Figure 15.

Figure 12 displays the PF maps illustrating the microstructural response of the joint corresponding to the crack tip at the AS. Figure 12b–d exhibit the microstructures at the SZ, RS, and AS, respectively. Upon observation, no discernible differences are evident in the microstructural morphologies compared to the joints before the FCG test shown in Figure 3. Consequently, the KAM maps were further analyzed. Figure 3 presents the KAM maps showing the microstructural response of the joint corresponding to the crack tip at the AS. Comparing with the results depicted in Figure 11, a significant release of residual strain is observed in the joint (i.e., the SZ, AS, and RS). Moreover, the residual strain release at the AS and RS is notably more pronounced, primarily concentrated prior to the FCG test.

Similarly, Figure 14 and Figure 15 illustrate the IPF maps and KAM maps, respectively, showing the microstructural response of the joint corresponding to the crack tip at the RS. Comparing these with the results depicted in Figure 13, it becomes apparent that the release of residual strain in the Str-CT-RS sample is more pronounced than that in the Str-CT-AS sample. This observation suggests that when the crack propagates at the AS, the residual strain in the joint is not fully relieved as it is at the RS. Consequently, the FCG rate of the Str-CT-AS sample is slightly higher than that of the Str-CT-RS sample.

### 4.3. Morphology of the Cracks

**Figure 16:**
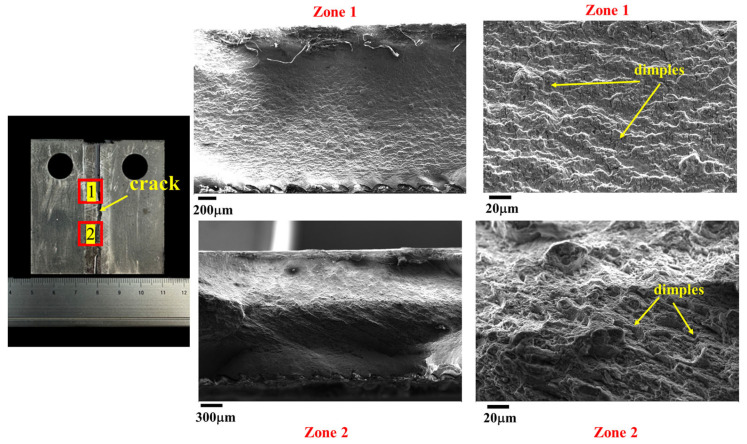
The morphologies of the crack at the SZ after the FCG test.

Figure 16 illustrates the morphologies of the crack at the SZ after the FCG test. The results reveal that the dimples of the fatigue crack passing through the SZ (i.e., zone 1 and zone 2) are shallow, consistent with the refined grains observed in the SZ. This finding aligns with the microstructural morphologies observed along the crack propagation path shown in Figure 8.

**Figure 17:**
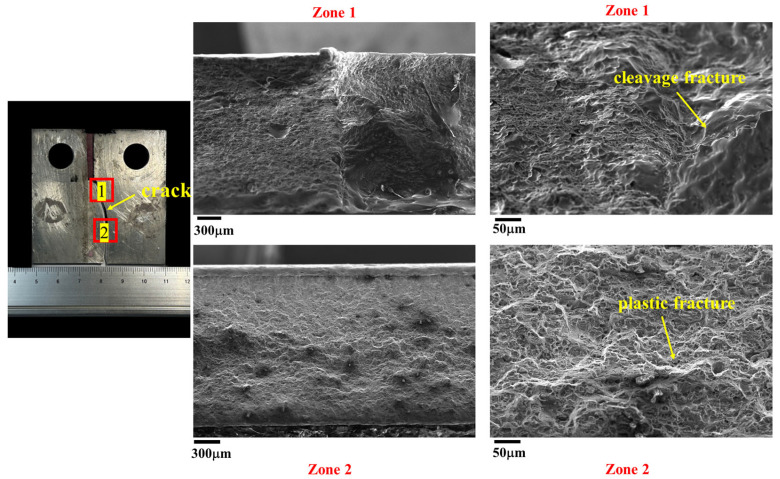
The morphologies of the crack at the AS after the FCG test.

**Figure 18:**
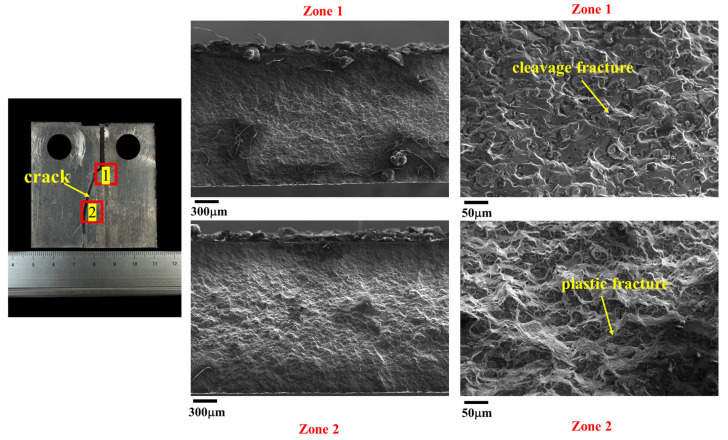
The morphologies of the crack at the RS after the FCG test.

Figure 17 and Figure 18 depict the morphologies of the crack at the AS and RS after the FCG test, respectively, both sharing similar features. The crack initiates at zone 1 and then gradually expands toward the BM, passing through zone 2. Consequently, zone 1 exhibits cleavage fracture morphology, while zone 2 shows plastic fracture characteristics. These results are in line with the microstructural morphologies observed along the crack propagation path shown in Figure 9 and Figure 10.

In summary, this study offers a quasi-in situ observation of the microstructural response during FCG of a friction stir welded AA2024-T4 joint, aiming to correlate fatigue crack growth behavior with mechanical properties investigated using EBSD. Therefore, in the future, the works that consider techniques such as in situ monitoring and digital image correlation to track FCG behavior in real-time are hoped to be conducted. Furthermore, multiscale computational models to simulate FCG behavior in FSW joints are hoped to be built based on this work to improve the reliability of friction stir welded joints during the performance.

## 5. Conclusions

The study focused on the quasi-in situ observation of the microstructural response during fatigue crack growth of friction stir welded AA2024-T4 joints to correlate FCG behavior with mechanical properties. Notched CT specimens corresponding to the SZ, AS, and RS were designed. FCG performances at different regions were obtained and characterized using the Paris model. EBSD was employed to examine the microstructure along the fatigue crack propagation path. The microstructural response of the joint during crack propagation at the AS and RS was compared to analyze differences in crack growth behavior between these regions. The following conclusions were drawn:

(1) The welding process improved the joint’s resistance to fatigue crack growth, with the base metal (CT-BM) exhibiting a shorter fatigue life compared to the welding zones (the SZ, AS, and RS). The SZ (Str-CT-SZ) demonstrated the highest fatigue load cycle. The FCG behaviors of the BM, SZ, AS, and RS were characterized using the Paris model.
(2) In the base metal, straight crack propagation characteristics were observed. In the SZ, cracks propagated through refined grains, contributing to the highest fatigue life and lowest FCG rate. Cracks initiated at the AS or RS and expanded towards the base metal.
(3) The phenomenon of the FCG rate of Str-CT-AS being higher than Str-CT-RS, despite similar microstructural features along the crack path at the AS and RS, was clarified. The significant release of residual strain in the Str-CT-RS sample compared to Str-CT-AS explained this difference. When the crack expanded at the AS, the residual strain in the joint was not fully relieved, leading to a slightly higher FCG rate in Str-CT-AS compared to Str-CT-RS.

## Figures and Tables

**Table 1:** Fitting results by Paris model in Figure 6.

Location	R^2^	*m*	lg*C*	*C*
BM	0.76	3.29	−6.98	1.05 × 10^−7^
SZ	0.77	5.79	−9.92	1.20 × 10^−10^
AS	0.69	4.98	−8.95	1.12 × 10^−9^
RS	0.65	5.25	−9.31	4.89 × 10^−10^

## Data Availability

Data are contained within the article.
